# Epicrania fugax with backward radiation: clinical characteristics of nine new cases

**DOI:** 10.1007/s10194-011-0353-1

**Published:** 2011-05-27

**Authors:** Patricia Mulero, Ángel L. Guerrero, Sonia Herrero-Velázquez, Elisa Cortijo, María Pedraza, María L. Peñas, Sara Miranda, Esther Rojo, Rosa Fernández

**Affiliations:** Department of Neurology, Hospital Clínico Universitario, Avda Ramón y Cajal 3, 47005 Valladolid, Spain

**Keywords:** Backward radiation, Epicrania, Epicrania fugax, Stabbing headache, SUNA, SUNCT

## Abstract

Epicrania fugax (EF) is a novel syndrome, described as a paroxysmal and brief head pain, starting in posterior cranial regions and rapidly spreading forward ipsilateral eye, nose or forehead. Two patients with comparable clinical features stemming from frontal scalp to ipsilateral posterior regions have been recently described and proposed as backward radiation epicrania fugax (BREF). We report a new series of nine BREF and compare their clinical characteristics with 18 forward radiation EF (FREF). Since first description of BREF in February 2010 we have assessed nine patients (four males, five females) with this clinical picture at an outpatient headache office in a Tertiary Hospital. Comparison is established with 18 FREF patients (6 males, 12 females), attended since the publication of first series of EF in March 2008. We found no differences between BREF and FREF, respectively, in age at onset (43.4 ± 13.1 vs. 42.5 ± 17.7 years), female/male ratio (5/4 vs. 12/6), pain intensity (6.9 ± 2.1 vs. 6.8 ± 2.1 in a 0–10 visual analogical scale), duration (7.1 ± 4.9 vs. 5.7 ± 4.3 s) and frequency of episodes per day (7 ± 8.4 vs. 9.9 ± 15.4). Patients in BREF group presented less frequently interictal pain in stemming point (22.2 vs. 55.5%) and accompanying autonomic signs (33.3 vs. 55.5%), but without statistical significance in both the cases. This series reinforces the proposal of EF as a new headache variant or a new headache syndrome. Clinical picture of brief pain paroxysms starting in the anterior scalp and radiating backwards does not fit known headaches or neuralgias and might correspond to a reverse variant of EF, clinical characteristics of which are comparable to FREF.

## Introduction

Epicrania fugax (EF) is a novel syndrome first described by Pareja et al. [1] in 2008 in ten patients that complained of a very brief unilateral pain paroxysms, starting in posterior cranial regions and rapidly spreading to ipsilateral eye, forehead or nose, along a linear or zigzag trajectory. In some cases, pain was accompanied by autonomic signs such as conjunctival injection, lacrimation or rhinorrhea.

To this first series, 15 new patients with the same clinical features have been added, so reinforcing the proposal of EF as a new headache variant or a new headache syndrome [2–4]. Recently, two patients who fulfilled all the characteristics for EF except the direction of radiation have been reported. This variant has been named backward radiation epicrania fugax (BREF) [5].

## Methods

Since the first description of BREF in February 2010, we made, in an outpatient headache clinic located in a Tertiary Hospital, a prospective search of the patients complaining of brief pain paroxysms running front to back, from anterior to posterior cephalic regions. As we searched for a novel headache syndrome, we took special care not to induce false answers during the interviews.

A detailed history was obtained in all the cases, including precipitant event and the coexistence of other types of headache. The characteristics of pain paroxysms were carefully evaluated, including temporal features (duration and frequency) and spatial features (site of origin, trajectory, and site of ending). Other characteristics such as pain quality, pain intensity (assessed with a visual analogical scale), or pain accompaniments, especially autonomic signs have been included. The presence of any triggers or interictal pain was also evaluated. Subsequently, a complete physical and neurological examination was performed, including inspection, palpation and sensory examination of the stemming area, as well as palpation of the supraorbital, infraorbital, supratrochlear, minor occipital and greater occipital nerves. Computed tomography or magnetic resonance imaging of the head, and routine blood work-up with erythrocyte sedimentation rate and immunological screening were carried out in all the cases.

We also made a prospective search of patients with a clinical picture from March 2008, which would be comprised under the headings or epicrania fugax with forward radiation, as was described by Pareja at al [1]. We registered all the demographic and clinical data of these patients. We included in the analysis characteristics of 6 patients previously described [2].

Statistical analysis was performed with SPSS 18.0 software. Level of significance was established at 5%. We compared the characteristics of both groups with a Chi-square test and also Fisher’s exact test, if necessary. Two-tailed Student *t* test was employed in the quantitative variables.

## Results

Through a one-year period, we assessed nine patients (four males, five females), with pain paroxysms comparable to BREF. Mean age at onset was 43.4 ± 13.1 years (range 26–62). Demographic and clinical features of these nine patients are shown in Table 1. Through a three-year period, 18 patients (6 males, 12 females) with FREF were attended in our headache clinic. Comparison of demographic and clinical characteristics in BREF and FREF patients is summarized in Table 2.

**Table 1:** Demographic and clinical features of nine BREF patients

Patient no.	1	2	3	4	5	6	7	8	9
Sex (M/F)	F	M	F	M	M	F	F	M	F
Age (years)	65	46	39	52	58	28	53	33	29
Age at onset	62	39	39	52	58	26	53	33	29
Other headaches	No	Migraine	Migraine	Tensión-type	Migraine	No	No	No	Migraine
Interictal Pain	No	No	No	No	No	Circumscribed	Circumscribed	No	No
Pain paroxysms									
Head side	Left	Right	Left	Left	Right	Left	Right	Right	Left
Site of origin	Eye	Forehead	Eye	Eye	Eye	Frontal	Frontal	Frontal	Eye
Site of end	Occipital	Occipital	Occipital	Occipital	Parietal	Parietal	Occipital	Occipital	Occipital
Trajectory	Linear	Linear	Linear	Linear	Linear	Linear	Linear	Linear	Linear
Character	Stabbing	Stabbing	Burning	Stabbing	Pressing	Stabbing	Electric	Stabbing	Stabbing
Duration (sec)	5	5	10	3	15	3	3	15	5
Intensity (VAS)	6	7	10	4	5	9	9	7	5
Accompaniments	No	No	No	No	Tearing itching eye	Rhinorrhoea itching eye	No	No	Itching eye
Triggers	No	No	No	No	Physical activity	Touch	No	No	No
Frequency	8/day	1–2/week	1/week	1/day	1/day	20/day	20/day	12/day	1/day
Temporal pattern	Chronic	Remitting	Remitting	Chronic	Remitting	Chronic	Chronic	Chronic	Chronic
Therapy	Amitriptyline	No	No	Amitriptyline	Gabapentin	Lamotrigine	Carbamazepine	Gabapentine	Gabapentine
Response	Complete			Complete	No	Complete	Complete	Partial	Partial

*M* male, *F* female, *VAS* visual analogical scale (0: no pain, 10: the worst imaginable pain)

**Table 2:** Main features of BREF and FREF patients

	BREF (*n* = 9)	FREF (*n* = 18)
Sex (F/M)	5/4	12/6
Age at onset (mean ± SD)	43.4 ± 13.1	42.5 ± 17.7
Duration sec (mean ± SD)	7.1 ± 4.9	5.7 ± 4.3
Frequency/day (mean ± SD)	7 ± 8.4	9.9 ± 15.4
Intensity VAS (mean ± SD)	6.9 ± 2.1	6.8 ± 2.1
Autonomic signs	3/9	10/18
Interictal pain	2/9	10/18
Triggers	2/9	4/18
Preventative requirement	7/9	12/18

*BREF* backward radiation epicrania fugax, *FREF* forward radiation epicrania fugax *M* Male, *F* Female, *SD* standard deviation, *Sec* seconds *VAS* visual analogical scale (0: no pain, 10: the worst imaginable pain)

We found no differences between BREF and FREF, respectively, in age at onset (43.4 ± 13.1 vs. 42.5 ± 17.7 years), and female/male ratio (5/4 vs. 12/6). A complete examination of stemming points did not reveal trophic changes in any of the patients and sensory disturbances were appreciated in 8 out of 12 patients, who suffered interictal pain. Palpation of pericranial nerves did not detect abnormal findings, and laboratory tests and imaging studies were also normal in all the cases.

Pain paroxysms were strictly unilateral in all BREF patients (in five cases on the left and in four on the right). Four cases described a history of migraine and one of tension-type headache, but EF was considered as quite different from the other headaches. Pain started in eye (*n* = 5), forehead (*n* = 1) or frontal region (*n* = 3), and inmediately spread backwards along a linear trajectory to occipital (*n* = 7) or parietal (*n* = 2) scalp. In two of the patients, there was an interictal pain in stemming point located in a circumscribed area, resembling nummular headache. In one of them exam disclosed a focal sensitive dysfunction. Paroxysms were described as stabbing in most of the patients (*n* = 6), or as electric, burning or pressing (1 patient each).

There was no significant difference between BREF and FREF, respectively, in paroxysms duration (7.1 ± 4.9 vs. 5.7 ± 4.3 s), pain intensity in a 0–10 visual analogical scale (6.9 ± 2.1 vs. 6.8 ± 2.1), frequency of episodes per day (7 ± 8.4 vs. 9.9 ± 15.4), presence of triggers (22.2 vs. 22.2%) and prophylactic treatment requirement (77.7 vs. 66.6%).

Patients in BREF group presented less frequently interictal pain in stemming point (22.2 vs. 55.5%) and accompanying autonomic signs (33.3 vs. 55.5%), but in both the cases without statistical significance.

## Discussion

In 2008, Pareja et al. [1] described a headache whose features did not fit any of the acknowledged headaches and they named it as epicrania fugax. This headache was framed within epicranias [6], a term proposed to group all the headaches that apparently stem from the superficial or extracranial structures, including the scalp and the layers of the skull. Common to this group of pain syndromes are a focal location or a sequence of multidirectional paroxysms, shortage of autonomic signs, and a dysesthesic area. Nummular headache is most typical epicranial disorder [7–9].

Regarding EF, there are several entities to which differential diagnosis have to be considered. For instances, there are similarities between EF and primary stabbing headache (PSH). PSH is a primary headache syndrome, with a female preponderance, characterized by short (3 s or less) stabbing pain paroxysms in a localized area that start and end in the same place [10, 11]. Frequency of paroxysms is highly variable and they usually occur with an irregular or sporadic temporal pattern [12]. Though the consecutive stabs may sporadically shift from one region to another giving an illusion of movement, clinical radiation observed in EF is definitely not typical for PSH [1].

Nummular headache (NH) resembles EF in the presence of a focal painful area. Pain in NH is typically continuous and, when paroxysms are superimposed, they begin and end in situ [9, 13]. Eight of our patients (two in BREF and six in FREF groups) had a nummular type head pain in the stemming area between EF paroxysms, as it has been previously described [3, 14]. EF and NH probably share a peripheral source in their pathogenesis [15].

Lacrimation, rhinorrhoea and itching eye are autonomic signs that appears in some of our patients. These signs are typical of other headache syndromes such as SUNCT (shortlasting, unilateral, neuralgiform headache attacks with conjunctival injection and tearing) or SUNA (shortlasting, unilateral, neuralgiform headache attacks with cranial autonomic features) [10]. These headaches are also present with painful neuralgiform attacks in the orbital and periorbital regions, triggered by tactile stimuli on trigeminal and extrageminal territories, and lasting from 2 s to 10 min. However, the dynamic component of pain, either posterior–anterior or anterior–posterior, in FREF and BREF are inherent qualities of epicrania fugax, not described in SUNCT or SUNA.

Unlike SUNCT, SUNA [5] can be diagnosed with the presence of just one cranial autonomic feature and the attacks have a wider range of duration. Thus, some of EF patients could be diagnosed of SUNA but the presence of a dynamic component of pain in EF is a quality not described for SUNA [16–18]. This is the essential attribute of EF: a fast and ample “movement” of the pain through one side of the head, no matter where the stemming point is.

The pathogenesis of EF is uncertain. Origin of this pain is probably peripheral according to the existence of a stemming or trigger zone in a focal area, and the stabbing or electric character of the pain. Paroxysms of pain can originate in the terminal branches of supraorbital nerve (SON) or greater occipital nerve (GON), and eventually extend to the peripheral fibers or branches. It has been proposed that the spreading of the pain is due to the electric transmission or paracrine diffusion of chemical mediators. Central mechanisms in which trigeminal afferents are implicated cannot be excluded, mainly in those patients presenting with autonomic signs [1].

## Conclusion

We report nine patients with the same features described for EF, except the direction of radiation. When we compare them with a group of patients fulfilling the characteristics of EF, we do not find differences between both the groups.

Our report reinforces EF as an independent headache syndrome and proposes BREF as a not infrequent variant of EF. Their features do not fit other acknowledged headaches or neuralgias. Another option is to in considered BREF and FREF as the same entity with the different presentations. Further observations are requiered for a definitive characterization of these headaches.
